# Perineural Administration of Botulinum Toxin for Chronic Pain Management: A Narrative Review

**DOI:** 10.1155/prm/6539538

**Published:** 2026-03-19

**Authors:** Théo Belaise, Mélanie Kremer, Bernard Poulain, Ipek Yalcin, Yohann Bohren

**Affiliations:** ^1^ Service D’anesthésie-Réanimation, Hôpitaux Universitaires De Strasbourg, Strasbourg, France, chru-strasbourg.fr; ^2^ Institut Des Neurosciences Cellulaires Et Intégratives (INCI), Centre National De La Recherche Scientifique (CNRS), Strasbourg, France, cnrs.fr; ^3^ Centre D’Evaluation Et Traitement De La Douleur, Hôpitaux Civils de Colmar, Colmar, France

**Keywords:** Botulinum toxin, neuropathic pain, perineural injection

## Abstract

**Background:**

Neuropathic pain occurs as a direct consequence of a lesion or a disease affecting the somatosensory system. Because of the limited therapeutic arsenal, it is difficult to treat. Nevertheless, pain relief has been demonstrated in multiple clinical trials treating neuropathic pain patients with subcutaneous or intradermal administrations of botulinum toxin. However, this type of administration route shows limits; for example, the painful area may be too large for subcutaneous administration, or administration discomfort would be intolerable for direct injection into the painful area. For these reasons, alternative routes have been investigated, including the perineural one.

**Objective:**

In the present review, we critically assess currently available clinical studies on perineural botulinum toxin administration for chronic pain treatment. We also discuss its possible underlying mechanisms and compare it to those of subcutaneous botulinum toxin administration.

**Results:**

Studies demonstrated that perineural injection of botulinum toxin is an interesting alternative for the treatment of chronic pain, both for reducing pain intensity and improving quality of life. In addition to few reported side effects, compared to other pharmacological interventions, its strength also lies in its long‐lasting effects.

**Conclusion:**

Despite promising results, the limited available literature cannot recommend this route of administration over others. More significant results and randomized controlled clinical trials are needed to support with confidence this route of administration.

## 1. Introduction

Neuropathic pain is defined by the International Association for the Study of Pain as *“pain resulting from injury or disease affecting the somatosensory system”* [1], a condition that decreases patients’ quality of life and represents a real socioeconomic burden [2–6]. Current guidelines recommend the use of antidepressants or anticonvulsants as first‐line treatment [7], despite their significant adverse effects and their poor therapeutic response: The number needed to treat (NNT) is 3.6, 6.4, and 7 for tricyclic antidepressants, serotonin and noradrenaline reuptake inhibitors, and gabapentinoids, respectively [8]. Among alternative therapeutic options, formulations containing botulinum neurotoxin (BoNT) type A (BoNT‐A) or type B (BoNT‐B) are one of the emerging treatments. These two serotypes (type A and B) share similar mechanisms of action but target different membrane proteins [9]. They can cleave distinct proteins of the soluble N‐ethylmaleimide‐sensitive factor attachment protein receptor complex (SNARE complex), implicated in the SNARE‐mediated vesicular release of neuropeptides as well as the membrane insertion of protein receptors at nerve endings [10, 11].

Recent clinical studies showed that subcutaneous injections of BoNT‐A can improve localized neuropathic pain symptoms [12–14], making BoNT a recommended treatment for neuropathic pain [7]. Experimental evidence supports the notion that subcutaneously injected neurotoxin is taken up by nociceptive nerve endings. This uptake is followed by inhibition of vesicular release of molecules involved in neurogenic inflammation, such as glutamate [15, 16], substance *p* (SP) [17, 18], and calcitonin gene‐related peptide (CGRP) [17, 19–23]. Additionally, it reduces SNARE‐mediated membrane insertion of receptors such as transient receptor potential vanilloid type 1 (TRPV1) [24–26] or purinergic receptor P2X ligand‐gated ion channel 3 (P2X3) [24, 27].

While all these mechanisms support the efficacy of BoNT‐A as a second‐line treatment for localized neuropathic pain [7, 8], multiple subcutaneous injections can be very painful [28–31]. To avoid multiple administrations in large areas already prone to allodynia or hyperalgesia, perineural administration of BoNT‐A around the nerve trunks or plexuses involved in chronic pain symptomatology has been proposed but remains poorly documented.

## 2. Method

The aim of this review is to summarize the literature on perineural administration of BoNT‐A in patients with chronic pain. The limited number of clinical studies and their heterogeneity precluded meta‐analysis. Therefore, we chose to present the data as a narrative review. As such, this work does not follow a predefined study selection or data extraction protocol, and its conclusions should be interpreted as exploratory and hypothesis‐generating, proposing an innovative concept for the use of perineural botulinum toxin in the management of neuropathic pain.

### 2.1. Search Methodology

A search of the MEDLINE database was conducted up to March 30, 2024, using the keywords “botulinum toxin AND pain,” which yielded the highest number of results. The first screening step excluded publications based on article type, species, and language. As a result, 2793 publications were removed, including reviews, books, studies involving nonhuman species, and non‐English‐language articles. The second step focused specifically on the use of perineural injections. To refine the search, additional terms such as “nerve block,” “neuropathic,” and “perineural” were used. This led to the exclusion of 828 more publications. Among the remaining 274 articles, a key inclusion criterion was that perineural injection sites had to be precisely guided using techniques such as ultrasound, fluoroscopy, or neurostimulation. A primary screening of titles and abstracts was followed by a full‐text review to verify that this criterion was met. Based on these selection criteria—and without excluding studies based on the origin of pain—a total of only 19 articles were ultimately selected and analyzed (Table 1). Limiting the search to MEDLINE and English‐language publications may have excluded relevant studies. However, given the exploratory, narrative nature of this review and the small number of available clinical trials, the impact of this limitation is likely minimal.

**TABLE 1:** Literature search strategy.

Identification	Electronic database search: MEDLINE Key words: « Botulinum toxin AND pain » 3895 hits	
Screening	‐ Type of article ‐ Species ‐ English language	Excluded (*n* = 2793): ‐ Reviews, systematic reviews, books ‐ Nonhuman studies ‐ Other languages than English
Eligibility (*n* = 1102)	‐ Target injection (using terms “nerve block” OR “neuropathic” OR “perineural”)	Excluded (*n* = 828) ‐ Other injection than perineural
Included	Studies assessing perineural botulinum toxin in pain conditions (*n* = 19)	Complex regional pain syndrome (*n* = 4) Peripheral neuropathic pain (*n* = 4) Abdominal or pelvic pain (*n* = 5) Chronic headache (*n* = 6)

### 2.2. Botulinum Toxin Administration

Different injection protocols and materials were used across etiologies (Table 2). BoNT‐A is available in several formulations with non‐equivalent dosing units, including onabotulinumtoxinA (Botox), incobotulinumtoxinA (Xeomin), and abobotulinumtoxinA (Dysport). In this review, most studies used onabotulinumtoxinA. One case report used incobotulinumtoxinA, while in another study the preparation was not specified. The administered doses ranged from 25 to 200 IU for onabotulinumtoxinA, 75–100 IU for incobotulinumtoxinA, and 5000 IU for botulinum toxin type B (Table 3).

**TABLE 2:** Etiologies of chronic pain and site of injection of perineural botulinum toxin.

Etiologies	Types of studies	Nerve target	Reference
Peripheral neuropathic pain	Case series	Peripheral nerves (radial, median…)	Meyer‐Frießem et al. 2019 [32]
	Case report	Brachial plexus; lumbar plexus	Moon et al. 2016 [33]
	Case report	Alveolar inferior and buccal nerves	Capon et al. 2022 [34]
	Case report	Femoral nerve; brachial plexus	Bohren and Timbolschi 2023 [35]

Complex regional pain syndrome	Randomized controlled trial	Lumbar sympathetic block	Yoo et al. 2022 [36]
	Randomized controlled trial	Lumbar sympathetic block	Carroll et al. 2009 [37]
	Retrospective	Lumbar sympathetic block	Lee et al. 2018 [38]
	Case report	Lumbar sympathetic block	Choi et al. 2015 [39]

Abdominal or pelvic pain	Randomized controlled trial	Scrotal cord block	Dockray et al. 2020 [40]
	Pilot study	Scrotal cord block	Khambati et al. 2014 [41]
	Case report	Impar ganglion	Lim et al. 2010 [42]
	Case report	Celiac plexus	Cho et al. 2020 [43]
	Case report	Celiac plexus	Sherman et al. 1995 [44]

Chronic headache	Randomized controlled trial	Grand occipital nerve	Ryu et al. 2019 [45]
	Case series	Grand occipital nerve	Kapural et al. 2007 [46]
	Randomized controlled trial	Sphenopalatine ganglion	Jamtøy et al. 2023 [47]
	Pilot study	Sphenopalatine ganglion	Yoshida 2020 [48]
	Pilot study	Sphenopalatine ganglion	Bratbak et al. 2016 [49]
	Pilot study	Sphenopalatine ganglion	Crespi et al. 2019 [50]

**TABLE 3:** Equivalence table between different types of botulinum toxin (adapted from Scaglione, 2016 [51]).

Botulinum toxin type	Brand name	Reported dose ranges	Dose equivalent units
Onabotulinumtoxin A	Botox	25–200 units	1
Incobotulinumtoxin A	Xeomin	75–100 units	1
Abobotulinumtoxin A	Dysport	Not applicable	2–3
Botulinum toxin type B	Myobloc/NeuroBloc	5000 units	BoNT‐B units are pharmacologically distinct from BoNT‐A and cannot be directly compared.

### 2.3. Treatment Response Assessment Modalities

The various studies most often assessed pain intensity as the main criterion, using the numerical scale or the Visual Analogue Scale (VAS). For cluster headache, one of the evaluation criteria was the number of attacks or pain frequency. Other secondary criteria, including patient satisfaction, using the Likert scale; the patient global impression of change (PGIC); and the impact of pain on quality of life, using the chronic epididymitis symptom index (CESI) or patient disability index (PDI) scores, were also used. These different assessments were carried out for time intervals ranging from a few days to several months post‐injection. Data related to the safety of perineural toxin injections were also collected and analyzed (Table 4).

**TABLE 4:** Summarizing study main outcomes of selected studies.

Etiologies	Type of studies	*n*	BoNT injection	Clinical pain outcome	Onset of action and duration	Adverse effect	Reference
Peripheral neuropathic pain	Retrospective study	60	‐ 1st injection: 25–100 IU onabotulinumtoxin‐A and ropivacaine 0.375% ‐ 2nd injection (*n* = 27/60): 25–100 UI onabotulinumtoxin‐A and ropivacaine 0.375% (average interval 127 ± 89 days)	−1 week (*n* = 60): 34 responders (mean pain reduction of 2.6 ± 1.6 NRS points; *p* < 0.001) and 26 nonresponders −3 months (*n* = 55): 50% of responder patients (17 of 34) reported a pain reduction. 20 patients declared to reduce pain medication.	Onset of action: < 1 W Duration: at least 3 M	Temporary weak paresis (3/60 patients). Undetectable after 8 weeks ‐ *n* = 2 at upper limb (ultrasound control injection) ‐ *n* = 1 at the levator labii superioris muscle following an injection at the trigeminal nerve (landmarks technique injection)	Meyer‐Frießem et al. 2019 [32]
	Case report	1	‐ 1^st^ injection: 25 IU onabotulinumtoxin‐A in saline ‐ 2^nd^ injection: 25 IU BoNT‐A in saline (2 weeks after the first injection)	−2 weeks: 25% of reduction for spontaneous and provoked pain. Improvement of global impression (PGIC). Apparition of persistent thermal chin allodynia. −3 months: pain had progressively returned to its initial stage	Onset of action: progressive apparition during 2 weeks. Duration: < 3 M	Transitory discomfort and flushing (1/1). Minimal labial paresis (no impact on speech or food intake).	Capon et al. 2022 [34]
	Case report	2	Injection: 50 UI onabotulinumtoxin‐A and 10 mL bupivacaine 0.1%	−4 months: pain relief with a VAS score from 9/10 to 2/10 (patient 2) −5 months: pain relief with a VAS score from 8/10 to 2–3/10 (patient 1)	Onset of action: < 1 M Duration: 4–5 M	None	Moon et al. 2016 [33]
	Case report	2	‐Injection: 75–100 IU incobotulinumtoxin‐A and 4 mL ropivacaine 0.2%	−1 month: pain relief with a VAS score from 7 to 9/10 to 3–4/10 (patient 1) −3 months: pain relief with a VAS score from 10/10 to 2/10 (patient 2)	Onset of action: ∼1 W Duration: 1–3 M	None	Bohren and Timbolschi 2023 [35]

Chronic headache	Randomized controlled double blind trial	54	‐Injection in control group (*n* = 27): 1 mL levobupivacaine 0.1% and 1 mg dexamethasone ‐Injection in BoNT group (*n* = 27): 50 IU onabotulinumtoxin‐A	−4 weeks: Pain reduction by comparison of VAS scores between the control group and the BoNT group (27.2 ± 4.1 vs. 13.9 ± 3.3 *p* < 0.05) −8 weeks: Pain reduction by comparison of VAS scores between the control group and the BoNT group (31 ± 4.2 vs. 9.3 ± 2.6 *p* < 0.05 −24 weeks: Pain reduction by comparison of VAS scores between the control group and the BoNT group (34.8 ± 5.8 vs. 12.3 ± 3.8 *p* < 0.05))	Onset of action: < 4 W Duration: at least 6 M	None	Ryu et al. 2019 [45]
	Case serie	6	‐Injection: 50 IU onabotulinumtoxin‐A	−4 weeks: Pain reduction with the VAS score from 8 ± 1.8 to 2 ± 2.7. Improvement of functional capacity with Pain Disability Index from 51.5 ± 17.6 to 19.5 ± 21	Onset of action: < 4 W Duration: average of 16.3 ± 3.2 weeks	None	Kapural et al. 2007 [46]
	Pilot study	10	‐Injection: 50 IU onabotulinumtoxin‐A in 1 mL saline using CAD/CAM‐derived injection guide	‐ VAS and pain frequency at baseline (8.1 ± 1.0 and 19.4 ± 8.8 times/day) was reduced: (*p* < 0.001) 2 weeks: VAS 3.5 ± 1.3 and 7.5 ± 5.5 times/day; 4 weeks: VAS 1.9 ± 1.4 and 4.8 ± 5.4 times/day; 8 weeks: VAS 1.9 ± 1.4 and 4.8 ± 5.5 times/day; 12 weeks: VAS 1.9 ± 1.4 and 4.8 ± 5.4 times/day	Onset of action: 2 W Duration: 12 W	None	Yoshida 2020 [48]
	Randomized, triple‐blind, placebo‐controlled, cross‐over study	30	‐ Injection in control group: 0.5 mL saline ‐ Injection in toxin group: 50 IU onabotulinumtoxin‐A in 0.5 mL saline	‐ between 5 and 8 weeks: pain severity showed no difference between BoNT and placebo (mean difference = 0.00, 95% CI, −0.57 to 0.57)	None	Diplopia (*n* = 5), 2 patients lasted for hours to days, and 3 lasted for 1–3 months. Facial asymmetry, 3 patients who experienced diplopia.	Jamtoy et al. 2023 [47]
	Pilot study	10	‐Injection: 0.05 mg adrenalin in 5 mL saline and 25 IU (*n* = 5) or 50 IU (*n* = 5) onabotulinumtoxin‐A using the MultiGuide®	−3 weeks and 4 weeks: diminution of the cluster headache attacks from 18 ± 12 per week in baseline to 11 ± 14 (*p* = 0.038) −6 months: reduction of cluster attack frequency for five out of 6 months post‐treatment	Onset of action: 3 W Duration: 6 M	Transient ipsilateral accommodation issues (*n* = 3) and chewing difficulties (*n* = 1) Severe adverse event: posterior epistaxis due to the transnasal procedure (*n* = 1)	Bratbak et al. 2016 [49]
	Pilot study	10	‐Injection: 25 UI onabotulinumtoxin‐A in 0.5 mL saline using the MultiGuide®	‐ Median number of attacks per day comparing baseline vs Weeks 5–8 was not statistically significant (*P* = 0.401). −4 patients were treatment responders with at least 50% reduction in the median number of attacks between baseline and Weeks 5–8, with 2 patients without attacks for 3 months	Onset of action: NS Duration: 3 M	Diplopia (*n* = 1) Nasolabial fold asymmetry (*n* = 2) Dysphagia resolved in 1 month (*n* = 1) Pain at the site of injection (*n* = 3)	Crespi et al. 2019 [50]

Complex Regional Pain Syndrome	Randomized controlled double‐blind trial	47	‐ Injection in control group (*n* = 24): 8 mL levobupivacaine 0.25% ‐ Injection in BoNT group (*n* = 23): 75 IU onabotulinumtoxin‐A	−1 month: Pain reduction with NRS scores in both groups compared to the baseline (−2.2 ± 1.0 for the toxin group and −1.0 ± 1.6 for the control group; *p* = 0.003) −3 months: Pain reduction with NRS scores in both groups compared to the baseline (−2.0 ± 1.0 for the toxin group and −0.6 ± 1.6 for the control group; *p* = 0.003) No pain medication reduction or modification of patient global impression	Onset of action < 1 M Duration: at least 3 M	Mild postprocedure dizziness (6/22 in control group and 3/22 in toxin group)	Yoo et al. 2022 [36]
	Randomized controlled double‐blind crossover trial	7	‐ 1^s^t injection (*n* = 7): 10 mL bupivacaine 0.5% ‐ 2^nd^ injection (*n* = 7): 75 IU onabotulinumtoxin‐A and 10 mL bupivacaine 0.5% (crossover after 1 month)	‐ BoNT was more effective at reducing VAS pain scores over time than bupivacaine alone (mean decrease VAS of 1.6; 95% CI 1.2–2.0; *p* < 0.0001). ‐ Days of analgesia: pain return was significantly lower after BoNT compared with local anesthetic alone: Median time to analgesic failure was 71 days after BoNT (95% CI, 12–253) compared to 10 days for local anesthetic alone (95% confidence interval, 0–12).	Onset of action: NS Duration: at least 2 M	Nausea and emesis resolved spontaneously after 2 days (1/9)	Carroll et al. 2009 [37]
	Retrospective	18	‐ Injection in BoNT‐A group (*n* = 5): 100 IU onabotulinumtoxin‐A ‐ Injection in BoNT‐B group (*n* = 13): 5000 IU BoNT‐B	−1 week: VAS scores were significantly lower in both groups compared to the baseline. ‐ Days of analgesia: Median time to return to baseline pain was 69 days in group B (95% CI, 45.5–92.5) compared to 15 days for group A (95% CI, 12.9–17.1).	Onset of action: 1 W Duration: 2 W (BoNT‐A) at least 2 M (BoNT‐B)	Transient dizziness (3/18) improved after 1 week.	Lee et al. 2018 [38]
	Case report	2	‐ Injection: 5000 IU BoNT‐B and 5 mL Levobupivacaine 0.25%	−2 months: pain decreased with VAS scores. Neuropathic symptoms with LANSS scores and vasomotor signs were improved	Onset of action: NS Duration: > 2 M	None	Choi et al. 2015 [39]

Abdominal or pelvic pain	Randomized, controlled, double‐blind trial	69	‐injection: 5 mL of a mixture of an equal combination of 1% lidocaine and 0.5% bupivacaine then 200 UI onabotulinumtoxin‐A and 10 mL saline for the toxin group and 10 saline alone for control group	−1 month: Mean VAS pain scores were not significantly changed after the blocks. The mean VAS score changed from 6.33 to 6.27 for total pain and from 6.53 to 6.03 for scrotal pain (*p* = 0.45 and 0.23, respectively)	None	None	Dockray et al. 2020 [40]
	Pilot study	18	‐ 1^st^ injection: diagnostic block with 10 mL bupivacaine 0.5% and lidocaine 2%. ‐ 2^nd^ injection: 100 IU onabotulinumtoxin‐A (average interval 152 days)	−1 month: Pain reduction with improved VAS scores compared to baseline for 13 patients (VAS average 7.36 vs. 5.61; *p* < 0.003) −3 months: 10 patients had lower VAS scores (VAS average 7.20 vs. 6.02; *p* < 0.05) −6 months: only 4 patients described an improvement in their VAS scores	Onset of action: NS Duration: < 6 M	Temporary transient scrotal/inguinal paresthesia (3/18). Resolved in all cases by 3 months.	Khambati et al. 2014 [41]
	Case report	1	‐ 1^st^ injection: 80 IU BoNT‐A and 1 mL bupivacaine 0.5% ‐ 2^nd^ injection: 100 IU BoNT‐A and 2 mL bupivacaine 0.5% (2 months after) ‐ 3^rd^ injection: 100 IU BoNT‐A and 2 mL bupivacaine 0.5% (6 months after)	−2 months: after the 1^st^ injection, VAS score decreased from 7 to 8/10 to 3/10 but increased after −6 months: after the 2^nd^ injection, VAS score decreased to 2/10 and PDI was improved	Onset of action: NS Duration: 2–6 M	None	Lim et al. 2010 [42]
	Case report	1	‐Injection: bupivacaine 5 mL 0.25% and 50 IU onabotulinumtoxin‐A mixed with 2 mL saline was injected on each side	−15 weeks: pain intensity was 0/10 on the VAS score and no medication for pain control	Onset of action: NS Duration: 15 W	None	Cho et al. 2020 [43]
	Case report	7	‐Injection: 1.5–2.5 UI/kg in 15 mL saline	‐Only one patient with a pain reduction. 2 patients with mild diminution for 5 and 8 days	Duration: 3 W	Transient nausea and emesis (1/7) Orthostatic hypotension (1/7)	Sherman et al. 1995 [44]

*Note:* LANSS, Leeds Assessment of Neuropathic Symptoms and Signs Pain Scale.

Abbreviations: CESI, chronic epididymitis symptom index; CI, confidence interval; M: month; NRS, Numerical Rating Scale; NS, nonspecified; PDI, patient disability index; VAS, Visual Analogue Scale; W, week.

## 3. Outcomes (Table 4)

### 3.1. Efficacy

Several studies analyzed show a reduction in pain intensity in patients after perineural injection of BoNT. This parameter was assessed using a numerical scale [32, 34, 36] or VAS. However, of these 19 studies, 7 are case reports and therefore describe analgesic effects [33–35, 39, 42, 43], except for one study, which reported pain relief in only one out of 7 patients with chronic pancreatitis‐related pain and a transient effect in two others [44].

For chronic regional pain syndrome, Yoo et al. [36] showed in a randomized controlled trial (RCT) that pain intensity was greatly reduced in the botulinum toxin group compared with the control group (local anesthetic alone) during the 3‐month follow‐up period (−2.0 ± 1.0 vs. −0.6 ± 1.6, respectively). The same conclusion was reported by Carroll et al. [37] in another RCT with a mean decrease in the VAS of 1.6 points in the botulinum toxin group (95% confidence interval: 1.2–2.0) and by Lee et al. [38] in a retrospective study with a median change in the VAS score of 2.5 points (range 1.0–6.0) compared to the preprocedure score.

In an RCT of headache patients, pain intensity (VAS score) was significantly lower at 4, 8, and 24 weeks post‐treatment in the BoNT‐A group (13.9 ± 3.3; 9.3 ± 2.6, and 12.3 ± 3.8) than in the control group (local anesthetic alone) (27.2 ± 4.1; 31.0 ± 4.2, and 34.8 ± 5.8) [45]. Interestingly, pain intensity was not different between the two groups 1 week after injection [45]. Moreover, a retrospective study also showed that pain relief after a BoNT‐A occipital nerve block lasted for at least 4 weeks, with a mean VAS score change from 8.0 ± 1.8 to 2.0 ± 2.7 [46]. In an RCT involving patients with persistent idiopathic facial pain, no significant difference was observed between BoNT‐A and placebo groups in terms of average pain during Weeks 5–8 following sphenopalatine ganglion (SPG) block. However, post hoc analyses suggest that the effect of BoNT‐A may occur within the first 4 weeks post‐injection, and a potential carry‐over effect in the cross‐over design may have led to an underestimation of BoNT‐A’s efficacy at later time points [47].

In a pilot study involving patients with trigeminal neuralgia, SPG block with botulinum toxin resulted in a decrease in both the VAS score (from 8.1 ± 1.0) and pain frequency (to 1.9 ± 1.4 and 4.8 ± 5.4 times/day, respectively) over a 12‐week period [48]. In another pilot study, the number of cluster headache attacks was significantly reduced from 18 ± 12 per week at baseline to 11 ± 14 in Weeks 3 and 4 (*p* = 0.038). The frequency of attacks remained significantly reduced for five out of 6 months post‐treatment [49]. In a further pilot study on refractory trigeminal neuralgia, the primary efficacy outcome was negative, with a median of 5.5 attacks per day at baseline compared to 5.0 during Weeks 5–8 (*p* = 0.401). However, four patients were considered responders, showing at least a 50% reduction in the median number of attacks between baseline and Weeks 5–8 [50].

In a prospective pilot study of pelvic pain, Khambati et al. [41] reported an improvement in pain intensity in 72% of patients (13/18) after BoNT‐A scrotal cord block. However, the change in the VAS score was relatively modest (mean VAS score: 7.36 before BoNT‐A vs. 5.61 after treatment). This limited difference may be explained by the fact that 56% of the patients experienced both scrotal and inguinal pain simultaneously, with minimal to no reduction in the overall VAS score, despite complete resolution of scrotal pain. In an RCT for scrotal pain, no statistically significant difference was observed in any measured outcomes between the BoNT‐A group and the controls. However, an open‐label extension was offered to men in the control group who continued to experience unchanged pain. In this group, 9 out of 13 men (69.2%) reported an improvement in VAS score (mean score reduced from 6.1 ± 1.66 to 4.5 ± 2.36, *p* = 0.022), with 6 of the 9 patients (66.7%) experiencing a reduction in persistent pain at 3 months [40]. These results suggest a potential placebo effect.

In a retrospective study of patients with peripheral neuropathic pain, responders were defined as patients with at least a 30% reduction in Numerical Rating Scale (NRS) score. Following perineural injection of BoNT‐A, 34 patients (57%) were responders with a mean pain reduction of 2.6 ± 1.6 NRS points. 50% of responders (17/34) reported pain relief for up to 3 months. Interestingly, 20 patients were able to reduce their pain medication. In contrast, nonresponders (26/60) showed no pain reduction or even a slight increase in pain intensity [32].

### 3.2. Quality of Life

Several studies have examined the effect of BoNT treatment on patient‐reported satisfaction (Likert satisfaction scale) [45], quality of life regarding pain using the CESI [41] or the PDI [42, 46], or daily life changes using the PGIC [34, 36]. Jamtoy et al. reported additional secondary outcomes, including quality of life assessed using various patient‐reported outcome measures collected at baseline and 12 weeks after each injection. These included the Chalder Fatigue Scale, the 36‐Item Short Form Survey, the McGill Pain Questionnaire, and the Hospital Anxiety and Depression Scale [47]. In studies reporting a therapeutic effect of botulinum toxin, the considered scores showed significant improvement, and pain intensity was reduced following the injections.

### 3.3. Time to Onset of Effect and Effect Duration

Few studies have intentionally recorded the time to onset of pain relief. The presence or absence of symptoms at the time of initial assessment (post‐injection) was the only indication providing information related to the onset time of effect. The latter varies from 15 days [34] to 1 month [41, 45, 46] for BoNT‐A administration. The time needed to return to initial pain levels after injection of BoNT‐A has been reported in one study [37]. One crossover study [37] and two RCTs directly compare the efficacy of local anesthetic ± steroids versus BoNT ± local anesthetic [36, 45]. Data show that the analgesic effect of perineural BoNT injection has a delayed onset (from several days to a week before onset) and a much longer duration compared to the analgesic effect of local anesthetic injection alone or in combination with corticoids [33, 42, 45].

When reported, median durations of pain relief were highly variable, ranging from 15 days to multiple weeks and up to 6 months [42] for BoNT‐A, while it was almost 2 months for BoNT‐B [38, 39].

### 3.4. Iterative Injections

Lim et al. [42] and Capon et al. [34] reported a reduction and attenuation of pain symptoms, such as allodynia, after repeated injections. However, spontaneous pain was reduced but still persisted after iterative injection [34]. Furthermore, Meyer‐Friessem’s work reported that repeated injections slightly increased the number of responders [32]. For example, of the four patients who had unsatisfactory pain relief after a first injection (< 30% reduction), one of them experienced pain reduction after an additional injection. In addition, patients who responded to the first injection continued to experience pain relief with subsequent injections. Thus, in 21 patients who had a satisfactory improvement in pain intensity (> 30% reduction), repeated injections of BoNT remained effective in the majority of them, that is, 18/21 (66.7%) [32]. In Sherman’s case report, for the responder patient, each new injection produced a similar effect lasting approximately 3 weeks, with no change in the duration of analgesia or pain intensity over time [44].

### 3.5. Safety

No serious adverse reactions threatening vital or functional prognosis were reported on this panel of articles. Only 30 out of 355 patients reported transient side effects, such as paresis, after a perineural injection of BoNT. In Meyer‐Friessem’s study [32], only one of the three cases of paresis reported was confirmed by clinical test. This involved the *levator labii superioris* muscle following an injection at the trigeminal nerve (injected by the landmarks technique). A blocking effect at the neuromuscular junction during the intramuscular passage of the needle seems to explain this paresis, rather than a direct perineural effect on the motor axon. This observation also seems valid for Capon’s case report [34]. SPG block procedures have been associated with adverse effects, particularly related to the intramuscular approach. In Jamtoy’s study, five patients developed diplopia—transient in two cases and lasting between 1 and 3 months in three cases, with facial asymmetry reported in the latter [47]. Additionally, 10 patients experienced procedure‐related pain requiring analgesics. In Bratbak’s study, three patients reported transient ipsilateral accommodation difficulties, and one experienced chewing problems; all resolved within 4 weeks. There was one severe adverse event: posterior epistaxis attributed to the transnasal procedure, independent of the toxin’s effect [49].

## 4. Discussion

BoNT is recognized as a second‐line treatment for localized neuropathic pain [7, 8]. Therefore, its subcutaneous administration should be considered for managing this type of pain. However, there are limitations to this approach. Indeed, the use of subcutaneous botulinum toxin in neuropathic pain is limited by the typically small size of the painful area (generally less than 300 cm^2^) and/or by technical difficulties, particularly when injections must be performed directly into a painful zone. In such cases, the perineural route may be considered a viable alternative, provided that the painful area corresponds to an identifiable nerve trunk or plexus. More broadly, perineural administration of botulinum toxin fits within the framework of peripheral neuromodulation strategies, alongside techniques such as pulsed radiofrequency [52] and cryoneurolysis [53]. To date, none of these techniques—cryoneurolysis, pulsed radiofrequency, or perineural botulinum toxin injection—has demonstrated clear superiority over the others. Moreover, due to a lack of sufficiently robust evidence in the literature, none of these methods is currently included in official guidelines for the management of neuropathic pain.

Most of the studies reviewed reported significant improvement in pain symptoms following perineural BoNT injections. Nevertheless, this narrative review has several limitations inherent to the heterogeneity of the available literature and the exploratory nature of the approach. The included studies relied on diverse methodological designs (RCTs, observational studies, and case series), exposing the findings to increased risks of selection bias and confounding. Study populations differed in clinical indications, patient characteristics, and inclusion criteria, limiting direct comparability across studies. Moreover, substantial variability was observed in intervention modalities, including the type of botulinum toxin used, dosing regimens, injection sites and techniques, pain etiologies, and outcome measures. The lack of protocol standardization and the frequently small sample sizes contribute to result imprecision and preclude quantitative synthesis or formal grading of evidence. Indeed, this review includes case reports, case series, and a limited number of RCTs spanning a wide range of chronic pain etiologies. A major limitation is the relatively small number of patients overall. The aim of this review is not to estimate treatment effects or formulate clinical recommendations, but rather to provide as comprehensive a synthesis as possible of the currently available data supporting the concept of perineural botulinum toxin as a potential therapeutic resource in neuropathic pain management. Accordingly, future research should prioritize comparative studies of dosing and injection protocols, as well as large‐scale, multicenter, long‐term, placebo‐controlled trials.

Nevertheless, this narrative review remains valuable in highlighting an innovative technique for botulinum toxin administration with encouraging clinical outcomes, often lasting several weeks or even months. However, some studies reported no improvement after one or more perineural injections, which may be attributable to inadequate dosing. To date, no study has clearly established optimal dosing regimens. Furthermore, given the non‐interchangeability of botulinum toxin units across formulations, dose comparisons between products should be interpreted with caution. Several studies show that the benefit of BoNT, although highly variable from patient to patient, appears to be dose‐dependent [41]. Some of the variability in BoNT‐induced pain relief may be related to the multiple etiologies of possible lesions or diseases of the somatosensory system at the origin of neuropathic pain [1, 54]. In fact, various clinical studies have identified different sensory profiles based on self‐questionnaires and clinical examination in populations suffering from chronic neuropathic pain of various etiologies [55–58]. However, there is currently no consensus among the various teams on the different sensory profiles that have been identified [55, 58]. One hypothesis is that the analgesic effect of the toxin varies with the sensory profile of chronic neuropathic pain. In this line, Attal et al. point out that the efficacy of BoNT by subcutaneous injection is greater in patients with mechanical allodynia or preserved thermo‐algesic sensitivity than in the other patients [12]. However, in their retrospective series, Meyer‐Frießem et al. did not identify any particular sensory profile of patients responding to perineural toxin injection [32]. A major difficulty in assessing the efficacy of a medication for chronic neuropathic pain is that studies include elements that relate not only to a medication’s ability to relieve pain but also to the context of the patient’s care.

Regarding headaches or facial pain, one of the underlying hypotheses involves the role of the autonomic nervous system in this type of disorder. Indeed, the pathophysiology is complex, involving all the somatic, trigeminal, and autonomic systems. Theories of cluster headache pathophysiology suggest the activation of a positive feedback loop, with a reflex arc comprising the trigeminal system as the afferent limb and the parasympathetic system—mainly via the SPG—as the efferent limb [59]. Therefore, the need for a diagnostic test block appears essential in identifying a relevant neural target prior to any potential neuromodulation with botulinum toxin. This step was not performed in the studies with SPG block [47–49]. Indeed, the accuracy of topographical diagnosis and the identification of underlying pathophysiological mechanisms are essential prerequisites for the effective management of chronic pain. Interventional pain management, in particular, requires a rigorous etiopathogenic approach aimed at identifying the pain‐generating mechanism responsible for the clinical symptoms. This process directly influences the relevance and appropriateness of the analgesic strategies employed. In this context, the use of peripheral nerve blocks for diagnostic purposes is of critical importance [60], as it enables confirmation of the peripheral origin of pain and provides a rational basis for the use of peripheral nerve modulation techniques.

In terms of safety, the current literature on the perineural route of BoNT administration does not highlight any serious adverse effects. This is consistent with the other routes of administration of the toxin used in other indications. In the recommendations of the Neuropathic Pain Special Interest Group (NeuPSIG), the safety of treating pain with BoNT is judged to be excellent for doses below 300 IU subcutaneous BoNT‐A per injection [8]. In comparison, patients with limb spasticity are injected intramuscularly with much higher doses of toxin: A European consensus established that a dose of about 600 IU of onabotulinumtoxin‐A and incobotulinumtoxin‐A or up to 1500 IU of abobotulinumtoxin‐A may be safe and well‐tolerated in adult spasticity [61]. Serious systemic side effects such as botulism‐like syndrome are most often related to inappropriate dose and frequency of reinjection, with the exception of anaphylactic reactions [61]. Injecting near nerve structures raises the question of potential neurotoxicity of BoNT. In this regard, a preclinical study showed a normal histology and neural architecture without cellular infiltration or demyelination after intraneural injection of BoNT [62]. Some rodent studies suggest that BoNT may facilitate the regeneration of nerve structures following injury [63–66].

**FIGURE 1:**
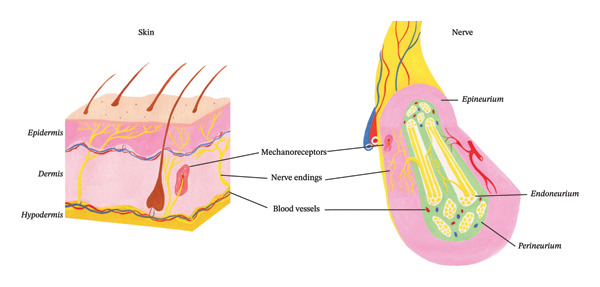
Illustration of the histology of a skin section and the various nerve endings and mechanoreceptors. These nerve endings are also known to be the entryway for botulinum toxin after subcutaneous administration. Based on the work of Hromada et al. [70], the nerve, like the skin, contains its own innervation called the nervi nervorum, consisting of nerve endings and mechanoreceptors. By analogy with the entry of botulinum toxin into the nerve terminals present in the skin followed by their retrograde transport to the sensory ganglia, the nerve endings in the nervi nervorum would mediate the entry of botulinum neurotoxin molecules applied in the perineurium into the nerve axons present in the nerve.

It is crucial to explore the site of action and the mechanisms that underlie the analgesic effect of perineural BoNT injections. Since BoNT inhibits SNARE‐mediated exocytosis [10, 11], the mechanisms underlying the potent painkilling effect of BoNT are likely related to the inhibition of the release of several mediators involved in the maintenance of chronic pain, such as glutamate, CGRP, SP, and the membrane availability of TRPV or P2X receptors [15, 23, 25, 67]. Thus, when it is injected subcutaneously, the toxin, thanks to its local action on nociceptive terminals and in the ganglia via retrograde axonal transport [68, 69], can modulate nociceptive message transmission at both the peripheral and ganglionic or even central levels. In addition, its action appears to induce a reduction in peripheral and central neuroinflammation [13, 17]. Next, the question arises as to the possible mechanism of action of BoNT when it is administered in the perineurium. This administration route raises a number of questions pertinent to its action, effect, and localization. Whether the toxin has a local or distal effect needs to be addressed. It could be transported orthogradely toward the nerve endings or retrogradely up to the dorsal root ganglion or trigeminal ganglion. However, the way it does penetrate the axons present in the nerve trunk and how it is sorted into vesicular cargoes that allow its transport is unknown. To the best of our knowledge, no studies have specifically investigated the intraneuronal or intraxonal internalization of BoNT after perineural application. J. Hromada’s work opens possible entryways. His/her group has shown around the nerve trunks, a complex net of blood vessels—the vasa vasorum—nerve processes, and terminals: the nervi nervorum [70, 71]. These nervi nervorum, mostly nonmyelinated, originate from nerve bundles and are located around the vasa vasorum and in the various layers of the nerve trunk (epineurium, perineurium, and endoneurium). These “nerve ending‐like” structures contain neuropeptides like SP and CGRP and are considered nociceptive endings [72, 73]. They might express BoNT receptors and therefore would be able to take up toxin molecules and sort them into transport vesicular cargoes, allowing their axonal transport. We propose that, like nociceptive nerve terminals in the skin, the nerve terminals of the nervi nervorum may serve as pathways for botulinum toxin to access nerve fibers that transmit pain signals when administered perineurally (Figure 1).

## 5. Conclusion

Clinical studies report significant and long‐lasting analgesic effects following perineural administration of BoNT in patients suffering from chronic pain. Despite their limited number and multiple biases, these studies support the hypothesis that perineural administration of BoNT could be proven complementary to its subcutaneous administration, depending on the anatomical surface involved, for neuropathic pain. With no additional risk compared to subcutaneous injection, BoNT strengthens its potential as a treatment candidate. The perineural administration studies open up interesting avenues for reflection, particularly on the feasibility, efficacy, and safety of such a technique, while keeping its use difficult to recommend for now. A more adequately powered and better‐designed RCT, such as the incoming PINBOT study, would allow for more definitive conclusions [74]. Finally, from a pathophysiological point of view, when applied via the perineural route, the questions regarding BoNT traffic and underlying action effects remain to be answered.

## Author Contributions

Théo Belaise and Yohann Bohren conceptualized the review, reviewed the literature, and wrote and revised the manuscript . Mélanie Kremer, Ipek Yalcin, and Bernard Poulain wrote and critically revised the manuscript.

## Funding

The authors have no sources of funding to declare for this article.

## Disclosure

All authors approved the final version to be published.

## Conflicts of Interest

The authors declare no conflicts of interest.

## Data Availability

Data sharing is not applicable to this article as no new data were created or analyzed in this study.

## References

[1] Scholz J. , Finnerup N. B. , Attal N. et al., Classification Committee of the Neuropathic Pain Special Interest Group (NeuPSIG). The IASP Classification of Chronic Pain for ICD–11: Chronic Neuropathic Pain, Pain. (2019) 160, no. 1, 53–59, 10.1097/j.pain.0000000000001365, 2-s2.0-85056353013. 30586071 PMC6310153

[2] Attal N. , Lanteri-Minet M. , Laurent B. , Fermanian J. , and Bouhassira D. , The Specific Disease Burden of Neuropathic Pain: Results of a French Nationwide Survey, Pain. (2011) 152, no. 12, 2836–2843, 10.1016/j.pain.2011.09.014, 2-s2.0-81055155921. 22019149

[3] Chenaf C. , Delorme J. , Delage N. , Ardid D. , Eschalier A. , and Authier N. , Prevalence of Chronic Pain With or Without Neuropathic Characteristics in France Using the Capture-Recapture Method: A Population-Based Study, Pain. (2018) 159, no. 11, 2394–2402, 10.1097/j.pain.0000000000001347, 2-s2.0-85055078707. 30028790

[4] Langley P. C. , Van Litsenburg C. , Cappelleri J. C. , and Carroll D. , The Burden Associated With Neuropathic Pain in Western Europe, Journal of Medical Economics. (2013) 16, no. 1, 85–95, 10.3111/13696998.2012.729548, 2-s2.0-84870894174. 22970839

[5] Critchlow S. , Hirst M. , Akehurst R. et al., A Systematic Review of Cost-Effectiveness Modeling of Pharmaceutical Therapies in Neuropathic Pain: Variation in Practice, Key Challenges, and Recommendations for the Future, Journal of Medical Economics. (2017) 20, no. 2, 129–139, 10.1080/13696998.2016.1229671, 2-s2.0-85010280479. 27563752

[6] Szewczyk A. , Jamroz-Wiśniewska A. , Haratym N. , and Rejdak K. , Neuropathic Pain and Chronic Pain as an Underestimated Interdisciplinary Problem, International Journal of Occupational Medicine and Environmental Health. (2022) 35, 10.13075/ijomeh.1896.01676. PMC10464730 35040826

[7] Moisset X. , Bouhassira D. , Avez Couturier J. et al., Pharmacological and Non-Pharmacological Treatments for Neuropathic Pain: Systematic Review and French Recommendations, Revue Neurologique. (2020) 176, no. 5, 325–352, 10.1016/j.neurol.2020.01.361. 32276788

[8] Finnerup N. B. , Attal N. , Haroutounian S. et al., Pharmacotherapy for Neuropathic Pain in Adults: A Systematic Review and Meta-Analysis, Lancet Neurology. (2015) 14, no. 2, 162–173, 10.1016/s1474-4422(14)70251-0, 2-s2.0-84922625305. 25575710 PMC4493167

[9] Rossetto O. , Pirazzini M. , and Montecucco C. , Botulinum Neurotoxins: Genetic, Structural and Mechanistic Insights, Nature Reviews Microbiology. (2014) 12, no. 8, 535–549, 10.1038/nrmicro3295, 2-s2.0-84904510042. 24975322

[10] Pirazzini M. , Rossetto O. , Eleopra R. , and Montecucco C. , Botulinum Neurotoxins: Biology, Pharmacology, and Toxicology, Pharmacological Reviews. (2017) 69, no. 2, 200–235, 10.1124/pr.116.012658, 2-s2.0-85016629565. 28356439 PMC5394922

[11] Poulain B. , Lemichez E. , and Popoff M. R. , Neuronal Selectivity of Botulinum Neurotoxins, Toxicon Avirulence. (2020) 178, 20–32, 10.1016/j.toxicon.2020.02.006. 32094099

[12] Attal N. , de Andrade D. C. , Adam F. et al., Safety and Efficacy of Repeated Injections of Botulinum Toxin a in Peripheral Neuropathic Pain (BOTNEP): A Randomised, Double-Blind, Placebo-Controlled Trial, Lancet Neurology. (2016) 15, no. 6, 555–565, 10.1016/s1474-4422(16)00017-x, 2-s2.0-84961233518. 26947719

[13] Matak I. , Bölcskei K. , Bach-Rojecky L. , and Helyes Z. , Mechanisms of Botulinum Toxin Type A Action on Pain, Toxins. (2019) 11, no. 8, 10.3390/toxins11080459, 2-s2.0-85071147498. PMC6723487 31387301

[14] Kim D. W. , Lee S. K. , and Ahnn J. , Botulinum Toxin as a Pain Killer: Players and Actions in Antinociception, Toxins. (2015) 7, no. 7, 2435–2453, 10.3390/toxins7072435, 2-s2.0-84937605169. 26134255 PMC4516922

[15] Cui M. , Khanijou S. , Rubino J. , and Aoki K. R. , Subcutaneous Administration of Botulinum Toxin A Reduces Formalin-Induced Pain, Pain. (2004) 107, no. 1-2, 125–133, 10.1016/j.pain.2003.10.008, 2-s2.0-0346094286. 14715398

[16] Bittencourt da Silva L. , Karshenas A. , Bach F. W. , Rasmussen S. , Arendt-Nielsen L. , and Gazerani P. , Blockade of Glutamate Release by Botulinum Neurotoxin Type A in Humans: A Dermal Microdialysis Study, Pain Research and Management. (2014) 19, no. 3, 126–132, 10.1155/2014/410415. 24851237 PMC4158957

[17] Lucioni A. , Bales G. T. , Lotan T. L. , McGehee D. S. , Cook S. P. , and Rapp D. E. , Botulinum Toxin Type A Inhibits Sensory Neuropeptide Release in Rat Bladder Models of Acute Injury and Chronic Inflammation, BJU International. (2008) 101, no. 3, 366–370, 10.1111/j.1464-410x.2007.07312.x, 2-s2.0-37849009378. 18184328

[18] Welch M. J. , Purkiss J. R. , and Foster K. A. , Sensitivity of Embryonic Rat Dorsal Root Ganglia Neurons to Clostridium Botulinum Neurotoxins, Toxicon. (2000) 38, no. 2, 245–258, 10.1016/s0041-0101(99)00153-1, 2-s2.0-0033991246. 10665805

[19] Durham P. L. , Cady R. , and Cady R. , Regulation of Calcitonin Gene-Related Peptide Secretion From Trigeminal Nerve Cells by Botulinum Toxin Type A: Implications for Migraine Therapy, Headache. (2004) 44, no. 1, 35–42, 10.1111/j.1526-4610.2004.04007.x, 2-s2.0-1642568305. 14979881

[20] Joussain C. , Le Coz O. , Pichugin A. et al., Botulinum Neurotoxin Light Chains Expressed by Defective Herpes Simplex Virus Type-1 Vectors Cleave SNARE Proteins and Inhibit CGRP Release in Rat Sensory Neurons, Toxins. (2019) 11, no. 2, 10.3390/toxins11020123, 2-s2.0-85061986615. PMC6409900 30791373

[21] Rapp D. E. , Turk K. W. , Bales G. T. , and Cook S. P. , Botulinum Toxin Type a Inhibits Calcitonin Gene-Related Peptide Release From Isolated Rat Bladder, Journal of Urology. (2006) 175, no. 3, 1138–1142, 10.1016/s0022-5347(05)00322-8, 2-s2.0-32044462256. 16469640

[22] Meng J. , Ovsepian S. V. , Wang J. et al., Activation of TRPV1 Mediates Calcitonin Gene-Related Peptide Release, Which Excites Trigeminal Sensory Neurons and is Attenuated by a Retargeted Botulinum Toxin With Anti-Nociceptive Potential, Journal of Neuroscience. (2009) 29, no. 15, 4981–4992, 10.1523/jneurosci.5490-08.2009, 2-s2.0-65549142642. 19369567 PMC6665337

[23] Meng J. , Wang J. , Lawrence G. , and Dolly J. O. , Synaptobrevin I Mediates Exocytosis of CGRP From Sensory Neurons and Inhibition by Botulinum Toxins Reflects Their Anti-Nociceptive Potential, Journal of Cell Science. (2007) 120, no. 16, 2864–2874, 10.1242/jcs.012211, 2-s2.0-34548567008. 17666428

[24] Apostolidis A. , Popat R. , Yiangou Y. et al., Decreased Sensory Receptors P2X3 and TRPV1 in Suburothelial Nerve Fibers Following Intradetrusor Injections of Botulinum Toxin for Human Detrusor Overactivity, Journal of Urology. (2005) 174, no. 3, 977–982, 10.1097/01.ju.0000169481.42259.54, 2-s2.0-23744514242. 16094018

[25] Shimizu T. , Shibata M. , Toriumi H. et al., Reduction of TRPV1 Expression in the Trigeminal System by Botulinum Neurotoxin Type-A, Neurobiology of Disease. (2012) 48, no. 3, 367–378, 10.1016/j.nbd.2012.07.010, 2-s2.0-84864921723. 22820141

[26] Matak I. , Rossetto O. , and Lacković Z. , Botulinum Toxin Type A Selectivity for Certain Types of Pain is Associated With Capsaicin-Sensitive Neurons, Pain. (2014) 155, no. 8, 1516–1526, 10.1016/j.pain.2014.04.027, 2-s2.0-84904569416. 24793910

[27] Xiao L. , Cheng J. , Dai J. , and Zhang D. , Botulinum Toxin Decreases Hyperalgesia and Inhibits P2X3 Receptor Over-Expression in Sensory Neurons Induced by Ventral Root Transection in Rats, Pain Medicine. (2011) 12, no. 9, 1385–1394, 10.1111/j.1526-4637.2011.01182.x, 2-s2.0-80052824923. 21810163

[28] Paracka L. , Kollewe K. , Wegner F. , and Dressler D. , Strategies to Decrease Injection Site Pain in Botulinum Toxin Therapy, Journal of Neural Transmission. (2017) 124, no. 10, 1213–1216, 10.1007/s00702-017-1764-1, 2-s2.0-85025599105. 28741118

[29] Le S. T. , Hanson C. , Rajpara A. N. , Liu D. Y. , and Aires D. J. , A Novel Anesthetic Technique for Palmar Botulinum Toxin Injection, Journal of the American Academy of Dermatology. (2017) 77, no. 2, e47-8–e48, 10.1016/j.jaad.2017.02.053, 2-s2.0-85030471876. 28711104

[30] Carruthers A. and Carruthers J. , Single-Center, Double-Blind, Randomized Study to Evaluate the Efficacy of 4% Lidocaine Cream Versus Vehicle Cream During Botulinum Toxin Type A Treatments, Dermatologic Surgery. (2005) 31, no. 12, 1655–1659, 10.2310/6350.2005.31304. 16336883

[31] Campanati A. , Lagalla G. , Penna L. , Gesuita R. , and Offidani A. , Local Neural Block at the Wrist for Treatment of Palmar Hyperhidrosis With Botulinum Toxin: Technical Improvements, Journal of the American Academy of Dermatology. (2004) 51, no. 3, 345–348, 10.1016/j.jaad.2003.09.006, 2-s2.0-4444369085. 15337974

[32] Meyer-Frießem C. H. , Eitner L. B. , Kaisler M. et al., Perineural Injection of Botulinum Toxin-A in Painful Peripheral Nerve Injury-A Case Series: Pain Relief, Safety, Sensory Profile and Sample Size Recommendation, Current Medical Research and Opinion. (2019) 35, no. 10, 1793–1803. 31148462 10.1080/03007995.2019.1626228

[33] Moon Y. E. , Choi J. H. , Park H. J. , Park J. H. , and Kim J. H. , Ultrasound-Guided Nerve Block With Botulinum Toxin Type A for Intractable Neuropathic Pain, Toxins. (2016) 8, no. 1, 10.3390/toxins8010018, 2-s2.0-84954450439. PMC4728540 26761032

[34] Capon C. , Crevant A. , Pointin A. , Sulukdjian A. , and Moreau N. , Botulinum Toxin A for Management of Refractory Concurrent Buccal and Inferior Alveolar Nerve Post-Traumatic Neuropathies: A Case Report, Journal of International Medical Research. (2022) 50, no. 9, 10.1177/03000605211047704. PMC9528003 36172992

[35] Bohren Y. and Timbolschi D. I. , Perineural Botulinum Toxin Injection for Cancer-Related Pain: Case Report of Two Patients, Pain medicine case reports. (2023) 7, no. 5, 261–264. 40929588

[36] Yoo Y. , Lee C. S. , Kim J. , Jo D. , and Moon J. Y. , Botulinum Toxin Type A for Lumbar Sympathetic Ganglion Block in Complex Regional Pain Syndrome: A Randomized Trial, Anesthesiology. (2022) 136, no. 2, 314–325, 10.1097/aln.0000000000004084. 34890455

[37] Carroll I. , Clark J. D. , and Mackey S. , Sympathetic Block With Botulinum Toxin to Treat Complex Regional Pain Syndrome, Annals of Neurology. (2009) 65, no. 3, 348–351, 10.1002/ana.21601, 2-s2.0-65249095310. 19334078 PMC2763598

[38] Lee Y. , Lee C. J. , Choi E. , Lee P. B. , Lee H. J. , and Nahm F. S. , Lumbar Sympathetic Block With Botulinum Toxin Type A and Type B for the Complex Regional Pain Syndrome, Toxins. (2018) 10, no. 4, 10.3390/toxins10040164, 2-s2.0-85047108004. PMC5923330 29671801

[39] Choi E. , Cho C. W. , Kim H. Y. , Lee P. B. , and Nahm F. S. , Lumbar Sympathetic Block With Botulinum Toxin Type B for Complex Regional Pain Syndrome: A Case Study, Pain Physician. (2015) 18, no. 5, E911–E916. 26431145

[40] Dockray J. , Aljumaily A. , Lau S. , and Jarvi K. A. , A Randomized, Double-Blind, Controlled Trial Shows That Onabotulinum Toxin A Nerve Blocks Do Not Provide Improved Pain Control in Men With Chronic Scrotal Pain, Journal of Urology. (2020) 203, no. 4, 767–772, 10.1097/ju.0000000000000658. 31738115

[41] Khambati A. , Lau S. , Gordon A. , and Jarvi K. A. , OnabotulinumtoxinA (Botox) Nerve Blocks Provide Durable Pain Relief for Men With Chronic Scrotal Pain: a Pilot Open-Label Trial, Journal of Sexual Medicine. (2014) 11, no. 12, 3072–3077, 10.1111/jsm.12707, 2-s2.0-84913615459. 25284738

[42] Lim S. J. , Park H. J. , Lee S. H. , and Moon D. E. , Ganglion Impar Block With Botulinum Toxin Type a for Chronic Perineal Pain-A Case report-, Korean Journal of Pain. (2010) 23, no. 1, 65–69, 10.3344/kjp.2010.23.1.65. 20552077 PMC2884216

[43] Cho N. R. , Kim Y. N. , Kim J. Y. et al., Celiac Plexus Block With Botulinum Toxin in Severe Chronic Pancreatitis—A Case Report, Journal of Clinical Pharmacy and Therapeutics. (2020) 45, no. 4, 848-51–851, 10.1111/jcpt.13180. 32437035

[44] Sherman S. , Kopecky K. K. , Brashear A. , and Lehman G. A. , Percutaneous Celiac Plexus Block WITH Botulinum Toxin A Did Not Help the Pain of Chronic Pancreatitis, Journal of Clinical Gastroenterology. (1995) 20, no. 4, 10.1097/00004836-199506000-00025, 2-s2.0-0029020250. 7665834

[45] Ryu J. H. , Shim J. H. , Yeom J. H. , Shin W. J. , Cho S. Y. , and Jeon W. J. , Ultrasound-Guided Greater Occipital Nerve Block With Botulinum Toxin for Patients With Chronic Headache in the Occipital Area: A Randomized Controlled Trial, Korean Journal of Anesthesiology. (2019) 72, no. 5, 479–485, 10.4097/kja.19145, 2-s2.0-85071184467. 31159537 PMC6781206

[46] Kapural L. , Stillman M. , Kapural M. , McIntyre P. , Guirgius M. , and Mekhail N. , Botulinum Toxin Occipital Nerve Block for the Treatment of Severe Occipital Neuralgia: A Case Series, Pain Practice. (2007) 7, no. 4, 337–340, 10.1111/j.1533-2500.2007.00150.x, 2-s2.0-36348972332. 17986166

[47] Jamtøy K. A. , Thorstensen W. M. , Stovner L. J. et al., Onabotulinum Toxin A Block of the Sphenopalatine Ganglion in Patients With Persistent Idiopathic Facial Pain: A Randomized, Triple-Blind, Placebo-Controlled, Exploratory, Cross-Over Study, Cephalalgia. (2023) 43, no. 7, 10.1177/03331024231187132. 37435807

[48] Yoshida K. , Sphenopalatine Ganglion Block With Botulinum Neurotoxin for Treating Trigeminal Neuralgia Using CAD/CAM–Derived Injection Guide, Journal of Oral and Facial Pain and Headache. (2020) 34, no. 2, 135–140, 10.11607/ofph.2510. 31560737

[49] Bratbak D. F. , Nordgård S. , Stovner L. J. et al., Pilot Study of Sphenopalatine Injection of onabotulinumtoxinA for the Treatment of Intractable Chronic Cluster Headache, Cephalalgia. (2016) 36, no. 6, 503–509, 10.1177/0333102415597891, 2-s2.0-84968813454. 26232105 PMC4853809

[50] Crespi J. , Bratbak D. , Dodick D. W. , Matharu M. , Jamtøy K. A. , and Tronvik E. , Pilot Study of Injection of OnabotulinumtoxinA Toward the Sphenopalatine Ganglion for the Treatment of Classical Trigeminal Neuralgia, Headache. (2019) 59, no. 8, 1229–1239, 10.1111/head.13608, 2-s2.0-85072362270. 31342515 PMC6771650

[51] Scaglione F. , Conversion Ratio Between Botox, Dysport, and Xeomin in Clinical Practice, Toxins. (2016) 8, no. 3, 10.3390/toxins8030065, 2-s2.0-84960080463. PMC4810210 26959061

[52] Chang M. C. , Efficacy of Pulsed Radiofrequency Stimulation in Patients With Peripheral Neuropathic Pain: A Narrative Review, Pain Physician. (2018) 21, no. 3, E225-34–E234, 10.36076/ppj.2018.3.e225. 29871378

[53] Biel E. , Aroke E. N. , Maye J. , and Zhang S. J. , The Applications of Cryoneurolysis for Acute and Chronic Pain Management, Pain Practice. (2023) 23, no. 2, 204–215, 10.1111/papr.13182. 36370129 PMC10107282

[54] Finnerup N. B. , Kuner R. , and Jensen T. S. , Neuropathic Pain: From Mechanisms to Treatment, Physiological Reviews. (2021) 101, no. 1, 259–301, 10.1152/physrev.00045.2019. 32584191

[55] Bouhassira D. , Branders S. , Attal N. et al., Stratification of Patients Based on the Neuropathic Pain Symptom Inventory: Development and Validation of a New Algorithm, Pain. (2021) 162, no. 4, 1038–1046, 10.1097/j.pain.0000000000002130. 33136982

[56] Baron R. , Dickenson A. H. , Calvo M. , Dib-Hajj S. D. , and Bennett D. L. , Maximizing Treatment Efficacy Through Patient Stratification in Neuropathic Pain Trials, Nature Reviews Neurology. (2023) 19, no. 1, 53–64, 10.1038/s41582-022-00741-7. 36400867

[57] Freeman R. , Baron R. , Bouhassira D. , Cabrera J. , and Emir B. , Sensory Profiles of Patients With Neuropathic Pain Based on the Neuropathic Pain Symptoms and Signs, Pain. (2014) 155, no. 2, 367–376, 10.1016/j.pain.2013.10.023, 2-s2.0-84892701578. 24472518

[58] Baron R. , Maier C. , Attal N. et al., German Neuropathic Pain Research Network (DFNS), and the EUROPAIN, and NEUROPAIN Consortia. Peripheral Neuropathic Pain: A Mechanism-Related Organizing Principle Based on Sensory Profiles, Pain. (2017) 158, no. 2, 261–272, 10.1097/j.pain.0000000000000753, 2-s2.0-85018196890. 27893485 PMC5266425

[59] Hoffmann J. and May A. , Diagnosis, Pathophysiology, and Management of Cluster Headache, Lancet Neurology. (2018) 17, no. 1, 75–83, 10.1016/s1474-4422(17)30405-2, 2-s2.0-85035142238. 29174963

[60] Hildebrandt J. , Relevance of Nerve Blocks in Treating and Diagnosing Low Back Pain--is the Quality Decisive?, Schmerz, Der. (2001) 15, no. 6, 474–483, 10.1007/pl00009801. 11793154

[61] Santamato A. , Safety and Efficacy of Incobotulinumtoxina as a Potential Treatment for Poststroke Spasticity, Neuropsychiatric Disease and Treatment. (2016) 12, 251–263, 10.2147/ndt.s86978, 2-s2.0-84957111214. 26869793 PMC4737345

[62] Lu L. , Atchabahian A. , Mackinnon S. E. , and Hunter D. A. , Nerve Injection Injury With Botulinum Toxin, Plastic and Reconstructive Surgery. (1998) 101, no. 7, 1875–1880, 10.1097/00006534-199806000-00015, 2-s2.0-0031777465. 9623830

[63] Cobianchi S. , Jaramillo J. , Luvisetto S. , Pavone F. , and Navarro X. , Botulinum Neurotoxin A Promotes Functional Recovery After Peripheral Nerve Injury by Increasing Regeneration of Myelinated Fibers, Neuroscience. (2017) 359, 82–91, 10.1016/j.neuroscience.2017.07.011, 2-s2.0-85026372429. 28716587

[64] Pamphlett R. , Early Terminal and Nodal Sprouting of Motor Axons After Botulinum Toxin, Journal of the Neurological Sciences. (1989) 92, no. 2-3, 181–192, 10.1016/0022-510x(89)90135-4, 2-s2.0-0024447480. 2809617

[65] Luvisetto S. , Botulinum Toxin and Neuronal Regeneration After Traumatic Injury of Central and Peripheral Nervous System, Toxins. (2020) 12, no. 7, 10.3390/toxins12070434. PMC7404966 32630737

[66] Marinelli S. , Luvisetto S. , Cobianchi S. et al., Botulinum Neurotoxin Type A Counteracts Neuropathic Pain and Facilitates Functional Recovery After Peripheral Nerve Injury in Animal Models, Neuroscience. (2010) 171, no. 1, 316–328, 10.1016/j.neuroscience.2010.08.067, 2-s2.0-77957870749. 20826198

[67] Zhang Y. , Lian Y. , Zhang H. , Xie N. , and Chen Y. , CGRP Plasma Levels Decrease in Classical Trigeminal Neuralgia Patients Treated With Botulinum Toxin Type A: A Pilot Study, Pain Medicine. (2020) 21, no. 8, 1611–1615, 10.1093/pm/pnaa028. 32167549

[68] Kitamura Y. , Matsuka Y. , Spigelman I. et al., Botulinum Toxin Type a (150 kDa) Decreases Exaggerated Neurotransmitter Release From Trigeminal Ganglion Neurons and Relieves Neuropathy Behaviors Induced by Infraorbital Nerve Constriction, Neuroscience. (2009) 159, no. 4, 1422–1429, 10.1016/j.neuroscience.2009.01.066, 2-s2.0-63349107908. 19409226

[69] Marinelli S. , Vacca V. , Ricordy R. et al., The Analgesic Effect on Neuropathic Pain of Retrogradely Transported Botulinum Neurotoxin A Involves Schwann Cells and Astrocytes, Public Library of Science ONE. (2012) 7, no. 10, 10.1371/journal.pone.0047977, 2-s2.0-84868109504. PMC3480491 23110146

[70] Hromada J. , On the Nerve Supply of the Connective Tissue of Some Peripheral Nervous System Components, Acta Anatomica. (1963) 55, no. 4, 343–351, 10.1159/000142483, 2-s2.0-84940143983. 14156453

[71] Bove G. M. and Light A. R. , The Nervi Nervorum, Pain Forum. (1997) 6, no. 3, 181–190, 10.1016/s1082-3174(97)70011-4.

[72] Bove G. M. and Light A. R. , Calcitonin Gene-Related Peptide and Peripherin Immunoreactivity in Nerve Sheaths, Somatosensory and Motor Research. (1995) 12, no. 1, 49–57, 10.3109/08990229509063141, 2-s2.0-0029165842. 7571942

[73] Appenzeller O. , Dhital K. K. , Cowen T. , and Burnstock G. , The Nerves to Blood Vessels Supplying Blood to Nerves: The Innervation of Vasa Nervorum, Brain Research. (1984) 304, no. 2, 383–386, 10.1016/0006-8993(84)90344-5, 2-s2.0-0021139108. 6204720

[74] Klee M. , Hørmann Thomsen T. , Enggaard T. P. et al., Perineural Injections of incobotulinumtoxin-A for Diabetic Neuropathic Pain of the Lower Extremities: Protocol for a Phase II, Single-Centre, Double-Blind, Randomised, Placebo-Controlled Trial (The PINBOT Study), BMJ Open. (2024) 14, no. 1, 10.1136/bmjopen-2023-074372. PMC10806716 38262642

